# Maternal mental health screening and management by health workers in southwestern Uganda: a qualitative analysis of knowledge, practices, and challenges

**DOI:** 10.1186/s12884-023-05763-7

**Published:** 2023-06-27

**Authors:** Gladys Nakidde, Edward Kumakech, John. F. Mugisha

**Affiliations:** 1 Department of Obstetrics and Gynecology, College of medicine, Pan African University of life and Earth Sciences Institute, University of Ibadan, Ibadan, Nigeria; 2 Department of Nursing, Faculty of Nursing and Health sciences, Bishop Stuart University, Mbarara, Uganda; 3 Department of Nursing and midwifery, Faculty health sciences, Lira University, Lira, Uganda; 4 Department of Health sciences, Faculty of Science and Technology, Cavendish University, Kampala, Uganda

**Keywords:** Maternal Mental problems, Puerperal psychosis, Maternal mental health, Sub-saharan Africa, Uganda, Maternal depression, Maternal anxiety, Maternal mental health

## Abstract

**Background:**

Maternal mental health (MMH) problems, such as perinatal depression, maternal anxiety, suicide ideation and puerperal psychosis among others, have a significant impact on maternal morbidity and mortality, as well as the health and development of children. One in every four pregnant women and one in every five postpartum women in low-income countries, suffer from maternal mental health (MMH) problems. Despite this, MMH screening, diagnosis, and reporting remain scanty in Uganda. Consequently, this study aimed to investigate the knowledge, practices, and impediments that maternity care workers face when screening and managing women with maternal mental health disorders in health facilities in south-western Uganda.

**Methods:**

In-depth interviews were conducted with 22 health-care professionals who work in maternity care departments in primary and tertiary healthcare facilities in southwestern Uganda to investigate their medical knowledge, clinical practices, and challenges related to the screening and management of maternal mental health problems. Using qualitative content analysis, distinct categories and subcategories were found.

**Results:**

Medical staff especially midwives lacked specialized training in screening and managing women with maternal mental health problems They screened and managed MMH problems solely based on history and physical examination, and they referred nearly every mother displaying signs of mental illness because they felt ill-prepared to handle them. On the other hand, medical staff with some level of specialized training in mental health particularly staff working in mental health units, were more likely to use a mental health screening tool in addition to history and physical examination; and to treat any women exhibiting signs and symptoms of maternal mental problems without referring them. Lack of in-service training on maternal mental health, poorly coordinated referral systems, reluctance of mentally ill to visit medical facilities, scarcity of mental health specialists, and shortage of relevant medications were identified as the major challenges. Age, experience level, or gender had no effect on screening or management practices.

**Conclusions:**

The results suggest that specialized training in mental health, and particularly maternal mental health, is essential for the effective screening and management of maternal mental health conditions in South Western Uganda.

## Background

Maternal mental health (MMH) illnesses during pregnancy and puerperium are major contributors to maternal morbidity and mortality, and have negative effects on the health and development of children [1–3]. MMH illnesses such as depression, anxiety, puerperal psychosis, are associated with adverse effects like feelings of self-harm (suicide ideations) or harm to the baby (infanticide), suicide, severe mood swings, loss of interest (apathy), disturbed sleep, loss of appetite and marital strife among many others [4–6]. Essentially, poor maternal mental health can substantially undermine the core of family stability and ultimately contribute to community disintegration.

In low- and middle-income countries (LMICs), including Uganda, one in four pregnant women and one in five postpartum women experience MMH problems respectively [7], indicating a high disease burden. A recent study [8] estimated postpartum depression at 27% in south western Uganda, but the overall MMH burden may be higher due to underreporting in maternity care settings [9, 10]. Premature births, living in a rural area, low self-esteem, and little access to social support, intimate partner violence, and low family income are among the risk factors for MMH problems that are highly prevalent in Uganda [8, 11–14]. In LMICs, the occurrence of MMH screening, diagnosis, and reporting is still very low [15–17]. This may be due to numerous factors such as lack of knowledge and skills by health workers to screen for MMH problems among others. For instance, a number of MMH screening tools such as the Edinburg postnatal depression scale (EPDS), Patient Health Questionnaire (PHQ-9) tool [11] for maternal depression and the Generalized Anxiety Disorder 7-item scale for anxiety, have been adapted in Uganda but their utility is more evident among specialized mental health workers and in research than in maternity care settings. During antepartum and postpartum period, women are usually attended to by midwives who mainly focus on physical pregnancy related concerns and pay little or no attention to MMH problems except in situations where mothers have severe mental health symptoms that require consultation or referral to mental health care specialists. The Uganda national mental health policy recognizes women with maternal mental illnesses as a vulnerable population, and as such, Uganda adopted the World Health Organization (WHO) initiative, the mental health gap action program (MHGAP), that aimed to train, equip and guide non-specialized health workers to screen and manage mild and moderate MMH problems [18, 19]. In Uganda, the mhGAP initiative was initiated in 2012–2016 as a pilot project to improve the well-being of people with mental health problems, including MMH, and reduce the treatment gap [11, 20]. However, previous research has shown that many MMH cases in maternity settings go unnoticed due to a focus on the physical needs of the mother and baby, high workloads, and a lack of training in MMH care knowledge and skills, resulting in delayed detection and care or missed diagnosis, which prolongs ill health and its consequences [11, 20, 21]. Therefore, this study explored how healthcare workers in southwestern Uganda screen for and manage MMH conditions, and the challenges they encounter.

## Methods

### Study design

This was a cross-sectional qualitative study.

### Study setting

The study was conducted at health centre (HC) III, IV and referral health facilities in two districts, that is Mbarara and Kabale, located in southwestern Uganda. The selected health facilities had maternity care facilities with antenatal and postnatal services. Southwestern Uganda is made up of 16 districts [22]. The two districts were purposively selected for the study due to the large number of health facilities available and the fact that they have regional referral hospitals, providing a larger catchment population [23].

In Uganda, provision of mental health services starts at health center III (HC III), that is a sub-county level, with subsequent referrals to health center IV (HC IV) at the county level, district hospitals, regional referral hospitals, and finally to the only national psychiatry referral hospital called Butabika Hospital, [24, 25]. From health center IV level upward, all cadres of medical professionals including mental health specialists (psychiatrists, psychiatric nurses and clinical officers) are available while HC III is headed by a diploma holder clinical officer or a diploma holder nurse, but without medical doctors (MDs/MBChB) and mental health specialists, offering outpatient clinic and maternity care services. Our study was conducted among health care workers from HC III level up to the regional referral hospitals within the selected districts namely Kabale and Mbarara district.

### Study population and sampling

Respondents to the study included specialized mental health workers (psychiatric nurses and psychiatric clinical officers) as well as non-specialized health professionals (midwives and nurses) who were directly involved in the screening and management of maternal mental disorders within the participating health care facilities, regardless of sexual orientation, age, or level of experience. Purposive sampling was used to ensure representation from the various categories of health professionals at the selected healthcare facilities in order to get insight into how various categories of health care professionals screened for and handled maternal mental health problems. A maximum of two health workers working in the maternal environment (antenatal or postnatal units) were purposively selected at each health facility (HC III and IV). Only individuals with at least two years of experience caring for pregnant or postpartum women or working as maternity unit managers (in-charges) knowledgeable with the clinical and administrative environments of the relevant health care facility were chosen. In addition to maternity care workers, specialized staff from the mental health department were included at the referral hospitals if they had a history of handling a woman with perinatal mental health difficulties. This purposive selection was bench marked on prior research [10] to capture rich experiences on how different types of health care workers screen for maternal mental health problems. A total of 22 respondents, including 10 from Mbarara district health facilities and 12 from Kabale district health facilities were recruited in the study.

### Inclusion criteria

The study included only specialized mental health workers (psychiatric nurses, psychotherapists, psychiatrists, and psychiatric clinical officers) and non-specialized health professionals (midwives and nurses) who were directly involved in the screening and management of maternal mental disorders within the participating health care facilities and who consented to participate, regardless of sexual orientation, age, or level of experience.

### Exclusion criteria

The study excluded all specialized mental health workers and non-specialized health professionals who were not directly involved in the screening and management of maternal mental disorders, did not consent to participate, or were absent at the time of data collection within the participating health care facilities.

### Data collection tools

We collected data through in-depth face-to-face interviews using open-ended questions designed in accordance to the study objectives. We developed the in-depth interview guide (Table 1) based on literature [10, 26] and consultation with experienced psychiatrists and mental health researchers. The interview guide explored basic screening and management skills and knowledge expected of a health care professional handling mothers with maternal mental illnesses, as well as challenges encountered when providing MMH services.

**Table 1:** The Interview guide used to get in-depth insight in how health workers assessed and diagnosed MMH problems

Biographic data: Job title, level of training, Gender, Age, Number of years in post, position held in the maternity care setting, designation (midwife, general nurse, mental health specialist etc.)
Please share with me if you had any training in mental health care (Probe: training when in school (pre-service)? any training in-service (post-school)? for how long?)
How confident are you to screen women with signs of mental illness in pregnancy and after child birth? (Probe which mental conditions can you screen for? Which mental conditions can you confidently manage?
How is screening and management of MMH problems done in the maternity setting at your facility? (Probe- how do you know that a woman has a MMH? Tell me more about how you screen at your facility; for depression; for anxiety. What about management?
How available and/or adequate are the care services for mental health during pregnancy and after child birth at your facility? (Probe- which care services for mental health exist at your facility? Which ones are missing?
What forms of chronic care arrangements exist in your maternity setting for MMH problems (probe- How are the victims followed up? –by phone, home visits, any support plans, SOPs for screening and management etc.
What are the challenges you encounter in providing chronic care to women with MMH problems in your maternity setting? (Probe- what may hinder you from screening or managing mothers with MMH
Kindly share with me the organizational support training avenues for MMH problems you have had in your maternity setting (probe- any planned continuous medical education (CME), or other training opportunities
What recommendations do you have for chronic care of women who suffer from MMH problems? (Probe- what should be done to ensure screening and management is done for women?
Give any more information that you think is relevant to improve MMH care in the maternity care settings

### Data collection procedure

The first author and trained research assistants conducted in-depth interviews between July and November 2022. There were two research teams each with two members. Each interview lasted approximately 30 to 45 min. All interviews were conducted in English, Uganda’s official national language. With the permission of each respondent, each interview was audio recorded and accompanied by field notes. Each study team interviewed a maximum of three respondents per day. One team member conducted the interview, while the other made notes and recorded the conversation with an audio recorder. With each subsequent interview, the team members reversed roles. We recruited respondents until saturation, or when no new information was gained from additional interviews [27]. Saturation was attained for specialized mental health workers at seven (7) and non-specialized health workers at fifteen (15). The interviews were conducted in a secluded room within the health care facility to ensure privacy, and each respondent was allocated a non-identifiable code rather than his or her name to preserve anonymity. Each respondent who agreed to participate in the study and for the audio recording was informed of their right to withdraw at any time without penalty.

### Quality control

The data collection research assistants (one medical doctor and three graduate nurses) were chosen based on previous experience in qualitative research as well as their baseline knowledge regarding mental health in their prior training and practice. The research assistants were trained for two days by the first author and an experienced mental health researcher on study objectives, informed consent, and data collection procedures, and then they participated in a hands-on pilot study, which increased their confidence in collecting data. Interview guides were field tested on three participants at a Health Centre III facility for practicality and intelligibility; however the results from the field test were not included in the final analysis. At the end of each day of data collecting, feedback meetings were held to review the data collecting process, discuss issues encountered, and plan for the next set of interviews. This ensured quality data and completeness. Completeness mainly centered on obtaining full information per question not necessarily the entire interview since participants were free to withdraw from the interview at any time. This was intended to obtain clarifications in time and in context before research assistants could forget and to perfect subsequent interviews. In order to guarantee that interviews were carried out correctly and in line with the study objectives, an experienced qualitative researcher provided oversight during the feedback process and did attend some of the interviews.

### Validity of the study

Trustworthiness including; credibility, transferability, dependability and conformability was ensured [28, 29].

#### Credibility

This was ensured by selecting only those respondents who met the inclusion criteria, and by strictly adhering to the in-depth interview guide. Furthermore, the research team ensured that the respondents correctly understood the questions and the research being conducted. We ensured that the respondents understood the study through respondent checks, and during the interviews, respondents were checked for understanding by rephrasing and summarizing the questions. We also carried out peer debriefing by reviewing the interview transcripts with the research team.

### Transferability

A purposive sample was used to enable researchers to maximize the range of information by consciously choosing respondents from different health facility levels and locations.

### Dependability

The researchers ensured detailed description of data collection methods to enable reproducibility. A consultant psychiatric clinician and researcher provided over sight by cross checking the data collected by the research teams to ensure that the information given by the respondents was accurately captured. The details of the interviews were recorded using a recorder, documented and verified.

### Confirmability

Confirmability was ensured by coding the information provided by the respondents and by ensuring that the interpretations of the data was not influenced by the researcher’s imagination [29]. The lead author carefully preserved all data for further analysis, and ensured use of verbatim respondent quotations to adequately demonstrate that the conclusions and their interpretation are supported by the data.

### Data management and analysis

Before proceeding to the next set of interviews, data was transcribed verbatim and cleaned. Data was transcribed in English because the respondents were all educated, that is, professional health workers, and so data was collected in English. The first author then checked the accuracy of the information against the audio recordings.

The data analysis was done using Creswell’s six-step model [27] which included; 1) organizing and preparing data for analysis through transcription, scanning through the transcripts; ii) reading transcripts to gain general understanding of the information and reflect on its meaning; iii) coding; iv) generate a description and categories and v) interpretation of findings.

The research team read and re-read the transcripts to become familiar with the data and ensure representativeness [30]. Each member of the research team independently created codes and categories from the data. The team then developed and jointly agreed on a coding framework. Each team member then independently coded a different transcript, and the coded work was later combined. The team assumed that each team member coded the work correctly since codes had been jointly established. Each team member conducted the initial analysis of the data, and shared and discussed the emerging categories with other authors. The unit of analysis was a transcript. Content analysis of the transcripts was performed using hybrid coding (both deductive and inductive) as initially, codes were developed from the data collection tool but additional categories and subcategories were included during analysis to capture the depth of the data. To highlight the most important aspects of the categories, respondents’ rich verbatim descriptions of emerging patterns in the data were used.

## Results

A total of 22 respondents were interviewed. Demographic characteristics are shown in Table 2.

**Table 2:** Characteristics of study respondents, n = 22

Characteristics of respondents	Frequency
Age in years;	28–52 years
Gender (%)	
Male Female	22 78
Education level (%)	
Certificate Diploma Degree	35 58 7
Designation (%)	
Midwives Doctors Psychiatric clinical officers and nurses who review women	68 6 27
Department (%)	
Antenatal (ANC) Postnatal (PNC) and young child clinic (YCC) All (ANC, PNC, YCC) Psychiatric department	9.0 23.0 41.0 27
Years of experience	5–20 years
District	
Kabale (n) Mbarara (n)	12 10
Health facility level (n)	
Health centre III Health centre IV Referral hospital	10 8 4

The study respondents were mainly midwives working in antenatal and postnatal departments. The respondents’ age range was 28–52 years, and their years of experience range was 5–20 years

From the analysis, hybrid coding (both deductive and inductive) was employed as initially, codes were developed based on literature review but additional categories and subcategories were included during analysis to capture the depth of the data. Three main categories are presented: (1) training and knowledge regarding maternal mental health; (2) screening and management of maternal mental health; and (3) Challenges in providing maternal mental health. Based on the common patterns that emerged from the aggregated data, additional subcategories were identified under the main categories namely: Training in MMH, Knowledge of MMH conditions, Confidence to screen and manage MMH problems, screening practices, management practices, challenges and recommendations.

### Category 1: Training and knowledge regarding maternal mental health

#### Subcategory 1: Training in maternal mental health

Apart from pre-service (in-school) training in general mental health, the vast majority of respondents claimed they had never taken any formal training on maternal mental health, and yet they indicated a desire to do so in order to provide better services.

“*Maternal mental health is something new and no health worker has formal training. If the training is available, we will be happy to participate and offer services because we are handling [women] as a whole. Mental health [training] was done in school as overall but not specific to maternal mental health. I have general knowledge on mental health”.* (Female, Midwife, 6 years work experience, non-specialized health worker).

Respondents who said they had ever received in-service mental health training said the focus was never on maternal mental health. Moreover, they stated that, when the opportunity arises, maternity care providers are not typically invited to such mental health trainings.

*I have had training in epilepsy management but not specifically in pregnancy during work (in-service). [Otherwise] I [last] had training in school generally.* (Female, Midwife, 4 year work experience, non-specialized health worker).

*When such opportunities come to train in mental health, they give chance to nurses but not us the midwives. So, we have never had any training, not even CME in mental health issues of mothers.* (Female, midwife, 10-year work experience, non-specialized health worker).

#### Subcategory 2: Knowledge of maternal mental health

The majority of respondents who worked in maternity units admitted to lacking the knowledge required to identify and treat pregnant women with mental illnesses. Some stated that they were able to detect symptoms of mental illness during pregnancy and after delivery, but they lacked the information or instructions needed to treat such women.

*I can identify some of the signs and symptoms [of MMH illness] and refer but I have no knowledge to manage the [women*]. (Female, Midwife, 10 years work experience, non-specialized health worker).

*There is no standard tool but in clerking this patient I am able to tell that the patient is mentally unwell.* (Female, midwife, 13 years work experience, non-specialized health worker)

### Category 2: Screening and management of maternal mental health

#### Subcategory 1: Confidence to screen and manage MMH problems

There were mixed responses when asked how confident they felt about screening and managing mothers with signs of maternal mental illness. In general, respondents with specialized mental health training reported higher levels of confidence than those without specialized mental health training. The majority of the latter expressed skepticism because they lacked knowledge of MMH as a discipline.

*I am somehow confident (to screen) of course not 100% but at least 70%. This is because of the tool, PHQ-9; I usually refer to during assessment.* (Female, Psychiatric nurse, 7 years work experience, specialized health worker)

*I can recognize signs and symptoms at least. I am somehow confident just that I lack the proper guidelines.* (Female, Midwife, 16-year work experience, non-specialized health worker).

#### Subcategory 2: Screening practices

When asked how they conducted screening and which screening tools they used to assess women with MMH illnesses, the majority of non-specialized respondents stated that they had no specific tools to assess and manage MMH illnesses aside from the standard clinical assessment methods of history and physical examination.

*“We have general form or questionnaires [Antenatal cards] that assess the mother’s health and history of mental health in her family. No guidelines for assessment of maternal mental health.”* (Female, Midwife, 6 years work experience, non-specialized health worker).

*“What appears on the antenatal care is the aspect of IPV [intimate partner violence] screening but from there the psychosocial assessment helps to bring out mental problems if present, but there is no specific screening tool to for depression or anxiety, though many mothers go without our notice.”* (Female, midwife, 18-year work experience, non-specialized health worker).

Those with specialized mental health training identified a number of tools for screening suspected patients for MMH disorders. Individuals and healthcare facilities used different tools, but the most notable were the Diagnostic and Statistical Manual of Mental Disorders (DSM-5), Mini Mental State Exam (MMSE) and Patient Health Questionnaire—9 (PHQ-9) screening tools.

“We *start at OPD [out patient department] with health education, we have a clinic every Tuesday where we sit with the patients and clerk them and see where the problem is. We have [use] DSM-V.”* (Male, Psychiatric nurse, 8 years of experience, specialized health worker).

*“When the midwives call us to review, we use the [mini] mental status exam and history to see if the mother has a problem… you can surely tell if the mother has psychosis or depression.*’’ (Female, psychiatric clinical officer, 18-year work experience, specialized health worker).

#### Subcategory 3: Management practices

The primary management strategy used by non-specialized respondents was to refer women with MMH illnesses to mental health specialists working at the same hospital (local referral) or at a different higher level care facility with specialized mental health care (external referral).

*“We screen them [mothers with signs of MMH illnesses] using general assessment and refer them [to psychiatrists] for management*.” (Female, Midwife, 13 years of work experience, non-specialized health worker).

*“…. But they may need more time to open up’’… Usually we refer them [to] the psychiatric team to help.” (Female, midwife, 18-year work experience, non-specialized health worker).*

The fact that screening and managing those mothers takes so much time is one of the factors cited for why non-specialist care providers refer mothers with MMH problems. Others cited a lack of knowledge of MMH.

### Category 3: Challenges and wayforward in providing mmh services

#### Subcategory 1: Challenges

When screening and managing women with MMH conditions, respondents mentioned a number of difficulties they encounter. These include;

##### Disjointed referral system

In most cases, the patients and their families are responsible for arranging their own transportation to the referral facility. As a result, a significant portion of referred mothers never receive the necessary medical care because they are unable to travel due to financial constraints.

*“……. a major challenge is the referral system in place. In case we get a [woman] with a mental challenge and needs referral, the referral system is poor, not in place, so the patient ends up going to higher.” (referral) facility on their own. Facility also has no transport (ambulance) so patients use private means”* (Female, Midwife,6 years work experience, non-specialized health worker).

##### Unfavorable community practices hindering seeking for health care

Another barrier mentioned was the community practice of hiding women who exhibit signs of mental illness. Respondents stated that this practice is most likely motivated by stigma and negative cultural perceptions of mental illness. The communities are not well sensitized about mental health. Because of the community negativity about mental health, many women are denied the opportunity to receive appropriate medical treatment, exacerbating their suffering.

*“Most mothers with maternal mental health problems are hidden by their families in the communities and do not come for treatment and it’s challenging to find them in community.”* (Female, Midwife, 18 years of work experience, non-specialized health worker).

*“………….. and communities are not sensitized on mental health.” (*Female, Psychiatric clinical officer, 7 years work experience, specialized health worker).

##### Lack of mental health care specialists and drugs for common MMH disorders

Respondents cited lack of mental health care specialists and drugs for the most common MMH disorders as among the challenges they faced. Because there are few psychiatric nurses, clinical officers, and doctors, consultation by non-specialized care providers is difficult. The number of patients is far too great in comparison to the number of available mental health specialists. The shortage of specialists is exacerbated by a lack of mental health specific medications in the majority of health care facilities that participated in the study. According to the respondents, maternity units are not supplied with antipsychotics because it is assumed that they only handle obstetric and gynecological conditions.

*“We don’t have psychiatric personnel so whenever we detect someone with mental health problems; we consult the ones in regional referral (hospital) which is hard. If we could get our own psychiatry personnel, it would be better. Also, there are no drugs for managing such conditions.” (*Midwife, 13 years work experience, non-specialized health worker)

##### Knowledge among non-specialized are providers was inadequate

A knowledge gap among non-specialist health care providers regarding MMH was also recognized among the key obstacles to provision of MMH services by respondents. The low level of training in mental health for the staff within the maternity care settings especially midwives who primarily receive, assess and manage pregnant and post-delivery mothers especially at lower-level facilities was a key challenge. Many midwives acknowledge a general lack of knowledge and hence confidence to manage MMH illnesses.

Most people running maternity department are enrolled midwives who have minimum capacity [training and knowledge] in managing these mothers.

(Female, Midwife, 12 years work experience, non-specialized health worker)

#### Subcategory 2: Respondents’ recommendations

Respondents made a number of suggestions when asked to offer possible solutions to the challenges faced in providing MMH services. One of the major recommendations was that MMH be given the same level of attention as other chronic conditions such as Human Immune Virus (HIV) in terms of raising awareness among health workers and the general public, as well as creating special clinic days with well-developed patient follow-up systems.

*“….there should be deliberate effort that all health care providers are reminded that these conditions exist in our community and be equipped enough so that they are able to identify these conditions early enough. Avail a clear appointment and follow-up of these patients like [is the case] with HIV*.” (Female, Midwife, 9 years work experience, non-specialized health worker).

Respondents also suggested providing pre-service (in school) and in-service maternal mental health training to midwives and other medical staff working in maternity units, in order for them to effectively recognize and treat women with MMH conditions without relying heavily on mental health specialists.

*“More trainings to the midwives to have the capacity to screen these mothers and also push for drugs used in treat of MMH problems.”* (Female, Midwife, 22-year work experience, non-specialized health worker).

*“I would love that as the midwives are employed, they should get refresher courses on maternal mental health.”* (Male, Psychiatric clinical officer, 10 years work experience, -specialized health worker).

Further recommendations from the respondents included the requirement for MMH screening tools to be developed and validated that are user-friendly for non-mental health professionals. The instrument needs to be incorporated into the postpartum and antepartum screening tools that are used regularly for women. This will make it possible to quickly identify women who are suffering from MMH conditions and provide them with the appropriate medical care.

*“We should have easy to use screening tools and this aspect integrated in the package offered to all pregnant mothers and post-delivery. …even where to document is very important for follow up*.” (Female, Midwife, 18 years work experience, non-specialized health worker).

Finally, in order to increase communities’ receptivity to MMH services on a cultural level, many respondents favored strategies by the government and relevant stakeholders to develop community outreach programs that involve communities in MMH awareness campaigns. They also advocated for properly stocking medical facilities with the right medications to treat MMH illnesses.

*“Government should also facilitate [healthcare] facilities with medication for MMH and resources, strengthen home visits and community outreaches to sensitize people”* (Female, Psychiatric nurse, 7 years work experience, specialized health worker).

## Discussion

This qualitative study investigated health care workers’ medical knowledge, clinical practices, and challenges in screening and managing maternal mental health services in southwestern Uganda. Respondents identified medical history, physical examination, and the use of tools such as the Diagnostic and Statistical Manual of Mental Disorders (DSM-5), Mini Mental State Exam (MMSE), and Patient Health Questionnaire—9 (PHQ-9) as the basis for assessing women with MMH problems. Screening and management of MMH conditions differed significantly between specialized mental health workers and other healthcare workers. Respondents with specialized mental health training were more likely to use standardized assessment tools or neuropsychological tests such as the MMSE, DSM-V, and PHQ-9. The provision of MMH services was hampered by disorganized referral systems, social norms that mothers showing signs of mental illness should be hidden, a lack of specialized mental health workers, a lack of medications tailored to MMH illnesses, and a knowledge gap among non-specialized healthcare professionals. Respondents noted that MMH screening and management services would be facilitated by community outreach programs to raise MMH awareness; in-service and in-school MMH training for all health workers, the development of screening tools that are user-friendly for non-specialized health workers, and an adequate supply of MMH-specific drugs in maternity units.

Our findings on health workers’ medical knowledge in relation to MMH reflected previous research claiming that specialized mental health workers who are relatively more knowledgeable in screening and managing MMH illnesses than their non-specialized counterparts are also very scarce [31, 32]. The problem of a shortage of specialists would be greatly alleviated by an effective system for referring patients to facilities with mental health specialists, but the results of our study showed that the referral system in the region is also inefficient. The disjoined referral systems are evidenced by family care takers having to organize independent transportation, being asked to fuel the ambulance and health workers not following up the victims to the referral center or even after. The poor referral system is also extensively reported in previous studies given that referrals lack professional escort, and referral centres don’t even receive prior communication from lower-level facilities yet still no feedback from the referral facility to the lower health facilities [33, 34]. A lack of clinician MMH knowledge, a scarcity of mental health specialists, and a poor referral system may explain why the recommended routine perinatal MMH screening [35–37] remains difficult, resulting in low levels of detection and management engagements of MMH problems in such regions [38]. A focus on maternal mental health is therefore necessary in the training of both mental health and non-mental health specialists, both in-service and in-school. Additionally, at policy level, it’s important to create strategies that promote deeper integration and cooperation between maternity care facilities and mental health care facilities.

In our study, only healthcare professionals who had received specialized training in mental health felt confident to screen and manage MMH conditions using a patient’s medical history and psychometric tests like the MMSE, DSM-V, and PHQ-9, whereas nearly all non-specialized care providers referred all women displaying signs of mental illness. The results of our study were supported by those of previous African studies [10, 27, 28, 31] that revealed a general lack of confidence among non-specialized health professionals to screen and treat specific mental health illnesses. Our findings appear to indicate that a sizable population of women in southwestern Uganda with MMH problems is likely to go undiagnosed due to the small number of healthcare professionals with mental health training [20], who are capable of using neuropsychological tests to screen for and treat specific mental illnesses [31]. Because effective screening is the foundation of effective care, all healthcare professionals should be trained on how to use the MMH screening tools that are currently available as part of any future interventions aimed at improving the quality of MMH care in southwest Uganda and other resource-limited settings [28]. It is also critical to consider how user-friendly the available screening tools are. The Edinburgh Postnatal Depression Scale (EPDS) for example, has been validated for use as a perinatal depression screening instrument in an African country (South Africa) with sensitivity of 0.80% and specificity of 76.6% [39]. However, in Uganda, there is unpublished work in the institutional online repository of Mbarara University of science and Technology on validation of the EPDS with psychometric properties of 86.8% sensitivity and 92.1% specificity at a cut off score of ≥ 10% [40] but its utility in clinical practice is limited [20], as none of the respondents in our study mentioned ever using them. This could be due to insufficient training in their use, or a lack of a mechanism for converting or applying research findings to practice [41, 42].

According to our study, one of the main challenges to MMH care services is the practice of women with MMH problems not seeking mental health care. This finding was consistent with the findings of numerous previous studies [20, 32, 43, 44]. Mothers may avoid seeking mental health care due to a lack of time for treatment, insufficient knowledge of available treatments, a lack of social support, unfriendly attitudes of health workers toward mental illness, and stigma associated with negative cultural beliefs about perinatal mental health illness [20, 32, 43, 44]. In many African societies, there are numerous misleading culture-based explanatory models that link maternal mental illness to vilifying notions such as failure to be a real mother, witchcraft, the influence of supernatural evil spirits, and the result of infidelity during pregnancy [11, 20, 45]. These perceptions have a substantial impact on the path of care, and as a consequence, several mothers with mental disorders may turn to prayers or traditional healers. Additionally, some women may choose to stay at home until the anticipated time of delivery to avoid the judgmental attitudes of medical professionals and the uncertainty of receiving support or assistance [44, 46–48]. Therefore, more research is needed into health workers’ attitudes, cultural beliefs, and social support structures for perinatal mothers with metal disorders in southwestern Uganda. Any interventions to improve MMH services must consider the influence of culture on health-seeking behaviors. It may be necessary to consider novel methods of providing MMH services to women in their homes, such as phone follow-ups (telemedicine) and home visits. Home visits more especially those initiated as early as the prenatal period have been recognized as effective avenues to improve perinatal mental health in a scoping review finding [49]. The review clarified that home visits offer an opportunity to detect and treat women and also reduce barriers to attaining mental health support for at risk women helping them to build self-confidence, and face uncertainties of new parenthood. Similarly, telemedicine has greatly evolved and has been recognized as an equally important approach to increase access to care and address health needs, including care for mental health especially in the error of staff shortages in low- and middle-income countries to effect home care and follow up activities [50]. Finally, our study findings agreed with previous research findings on the issue of maternity unit shortages of mental health specific medications [20]. Factors contributing to this observation may include a total lack of supply or insufficient supply by the government, or a policy directive based on the mhGAP program’s recommendation to limit the use of antipsychotics in perinatal periods in favor of non-pharmacological treatments (psychotherapy) like group therapy, individual therapy which involve talking sessions with the patient [19]. It is noteworthy that despite the program recommendation to opt for non-pharmacological therapies as first line during pregnancy and postpartum, this is less practiced since majority of respondents mainly focused on drug scarcity as a challenge to managing cases identified. Insufficient knowledge of available treatments reported by some respondents could have contributed to this phenomenon as also spelt out by Daehn et al. [51] and [11]. Therefore, there should be a ministry of health and health facility management initiative to have more awareness campaigns be put in place to ensure health care workers are updated on drug choices, dosages as well as the first line non-pharmacological interventions appropriate for antepartum and postpartum women with mental health problems so as to minimize drug related negative consequences during this critical period [19]. In addition, adequate supply of drugs by relevant drug authorities, and enhancing a closer cooperation between maternity centers and mental health care facilities within hospitals and across regions may aid in overcoming drug shortages and the treatment gap challenge.

### Study strengths and limitations

Individual interviews and qualitative content analysis were used in the design of this qualitative study. Since the scope of individual interviews is limited, it is challenging to make meaningful statistical inferences and extrapolate the results to other contexts. To get a deep understanding of experiences and beliefs connected to a phenomenon, however, individual interviews and qualitative content analysis are most effective [52, 53]. As a result, the findings from this study offers a thorough understanding of the skills, routines, and difficulties faced by medical professionals in southwestern Uganda when screening and managing perinatal mothers with mental health disorders. Furthermore, our data lay the groundwork for future interventional research projects aiming to enhance MMH services in Uganda and LMICs, as well as opportunities for future population studies to quantify the MMH burden in Uganda.

## Conclusion

Our study revealed patient, social, and health worker-related barriers to MMH service provision, warranting intervention and policy approaches that effect change at multiple levels, namely, increasing health worker knowledge of MMH, improving closer cooperation and integration of MMH services with general mental health care provision, and increasing MMH awareness in communities. More research is required to examine the impact of health workers’ attitudes, cultural beliefs, and social support networks on MMH care services.

## Data Availability

The dataset for the current study not publicly available due confidentiality restrictions, but is available from the corresponding author on reasonable request.

## Abbreviations

MMH: Maternal Mental Health Problems
WHO: World Health Organization
ANC: Antenatal care
PNC: Postnatal care
